# The effect of nutrition, hatching time, and hatching conditions on the weight gain and survival of ostrich (*Struthio camelus*) chicks until 4 weeks of age

**DOI:** 10.1007/s11250-026-05114-6

**Published:** 2026-06-04

**Authors:** Dóra Lili Brassó, István Komlósi, Béla Béri, Levente Czeglédi, Zsolt Somogyi, Zsófia Várszegi

**Affiliations:** Department of Animal Science, Institute of Animal Science, Biotechnology and Nature Conservation, Faculty of Agricultural and Food Sciences and Environmental Management, University of Debrecen, Böszörményi Street 138, Debrecen, 4032 Hungary

**Keywords:** Starter feed efficiency, Early nutrition, Ostrich weight gain, Chick mortality, Hatching conditions

## Abstract

The study investigated how three types of commercial starter diets, formulated for ostrich (OS), turkey (TS), and chicken (CS), along with hatching time and manual assistance, affected the early growth and survival of ostrich (*Struthio camelus*) chicks during the first 4 weeks of rearing. A total of 75 chicks were individually marked and randomly assigned to one of the three diet groups. From day 5, chicks received a 55% starter feed and 45% alfalfa hay mix *ad libitum*. Weekly body weight and daily mortality were recorded. Based on hatching circumstances, chicks were categorized as unassisted on day 40 (D40), assisted on day 41 (D41), or assisted on day 42 (D42). We applied univariate analysis of variance (GLM) for weekly weight and weight gain analysis, and we investigated the correlations between hatching group and hatching weight, and hatching group and 4-week growth intensity with Pearson’s bivariate correlation in SPSS 22.0. We performed Kaplan–Meier procedure and Cox regression in SPSS 22.0 to analyse the effect of hatching and feeding groups on the relative culling risk of chicks. Results showed that the starter diet strongly influenced both growth and survival. Starter feed with 11.4 MJ/kg metabolizable energy, 25.91% crude protein, and 3.89% crude fiber content showed the best effects both on growth intensity (341.41 g in weekly average) and survival (85%) during the 4-week experimental period. Chicks fed with starter feed with similar energy (11.1 MJ/kg) and similarly low (5.08%) crude fibre, however, remarkably lower crude protein (17.9%) content performed moderately well (239.79 g in weekly average with 76% survival). In contrast, starter feed with low metabolizable energy of 8 MJ/kg, relatively low (17.77%) crude protein, but extremely high crude fibre (13.00%) content resulted in poor growth (193.34 g in weekly average) and extremely high mortality (> 80%), with only 16% surviving. Hatching groups also exhibited significant differences in chick mortality, with D40 showing the lowest culling risk. These findings highlight that early nutrition is critical, and starter diets with higher metabolizable energy and protein and lower crude fiber markedly improve survival and productivity in ostrich chick rearing.

## Introduction

Ostriches were domesticated in South Africa in the 19th century for feathers, leather, and meat production (Engelbrecht 2013). Ostrich meat is a red meat known for its low intramuscular fat and high antioxidant content (Adams and Revell 1998). Today, major ostrich industries operate in China, Brazil, South Africa, and Pakistan, while the highest populations in the EU are in Ukraine, Romania, Poland, and Germany (Kistner 2019).

The growth and development of ostriches, particularly at young ages, hold economic significance. Growth intensity depends on several factors, primarily feeding and management. The nutritional needs of young birds are determined by their weight gain and the energy content of the diet (Miah et al. 2020). Compared to poultry and swine, research on precise ostrich nutrition remains limited (Mushi et al. 1998; Tasirnafas et al. 2015). Few studies have analysed chick weight gains and performance in relation to varying feed compositions, and existing recommendations for optimal energy and crude protein levels remain inconsistent (Aganga et al. 2003; Cilliers 2000; Miao et al. 2003). Mortality rates in ostrich chicks under 3 months can reach 50% (Smith et al. 1995). Chicks are highly sensitive to environmental factors, including nutrition, stress, hygiene, and stocking density (Elobeid et al. 2014), particularly between 1 week and 2 months of age (Adewumi et al. 2017). Malnutrition and imbalanced diets pose significant risks, emphasizing the need for optimal levels of energy, protein, fiber, vitamins, and minerals (Awalom 2023; Cooper 2000).

Research on optimal feeding strategies is limited and often inconsistent. Additionally, knowledge on the effects of hatching time and conditions on ostrich chick development and mortality is also scarce. This study evaluated the effects of three commonly used starter diets (turkey, chicken, and ostrich), differing in nutritional composition, on the weight gain and survival of ostrich chicks up to 4 weeks of age. We hypothesized that diets with higher metabolizable energy and protein content would improve early growth and survival, and assisted hatching would be linked to poorer outcomes. The results provide preliminary insights into practical ostrich nutrition and its implications for chick development and production efficiency.

## Materials and methods

### Study population and husbandry conditions

We conducted the experiment from August 20 to September 12, 2025, on a large ostrich farm in Hajdú-Bihar County, East Hungary, which housed 130 breeding birds. The farm raised chicks until they reached 4 weeks of age before exporting or selling them to local breeders for fattening. The chicks used in this study hatched between August 15 and August 17, 2025. We incubated all eggs in the same incubator under identical conditions.

We included a total of 75 individually identified chicks, tagged with radio frequency identification (RFID) microchips, in the analysis. The tags were used only for individual identification. Five days after hatching, we selected clinically healthy chicks of unknown genotypes and transferred them to a battery cage (2.5 m × 1.5 m) with a stocking density of 11 chicks/m², where they had access only to water. On day 5, we randomly divided the chicks into three experimental groups and moved them to a barn with a concrete floor and an adjacent paddock.

We ensured that all 3 groups experienced the same environmental conditions by separating designated spaces within the barn and paddock. Each paddock measured 70 m², with half of the area covered with concrete and the other half with sand. The indoor barn space was 14 m², where we housed the chicks at night and during cold weather. Each group had a stocking density of 0.33 chicks/m². We maintained the barn temperature between 28 °C and 30 °C using 2 infrared lamps per group. We provided water and feed using gravitational drinkers and wooden feeders. We sanitized both the barn floor and paddock daily with a chloride solution to maintain hygiene.

### Description of the feeding experiment

We fed the chicks for the first time at 5 days of age. The diet consisted of 55% starter feed and 45% alfalfa hay, offered *ad libitum* throughout the experiment. We assigned the 3 feeding groups to receive either turkey, broiler chicken, or ostrich starter diets, and placed 25 chicks in each group. So, altogether 75 chicks were involved in the analyses without repetition of the groups.

### The composition of starter diets

*Turkey starter (TS)*: soybean meal, wheat, corn, barley, DDGS (distillers dried grains with solubles), CGF (corn gluten feed), rye, corn germ, sunflower meal, lime, MCP (monocalcium phosphate), salt, and sodium bicarbonate.

*Chicken starter (CS)*: wheat, wheat meal, corn, barley, CGF, DDGS, soybean meal, corn germ, wheat bran, rye, lime, and salt.

*Ostrich starter (OS)*: barley, alfalfa pellet, wheat, extracted sunflower meal, dried beet pulp, extracted soybean meal, corn, extruded sunflower seeds, MCP, 1.5% ostrich starter premix, lime, sunflower oil, and antioxidant (E321).

There were great differences in the analytical composition of starter diets (Table 1).

**Table 1:** The analytical composition of starter feeds^1^

Type of starter feeds			
Composition	Ostrich starter (OS)	Turkey starter (TS)	Chicken starter (CS)
ME^2^ (MJ/kg)	8.00	11.4	11.1
Dry matter (%)	88.10	88.28	87.88
Crude protein (%)	17.77	25.91	17.90
Crude fat (%)	3.23	2.99	2.71
Crude fiber (%)	13.00	3.89	5.08
Ash (%)	7.10	6.69	4.99
Lysine (%)	1.06	1.49	0.82
Methionine (%)	0.60	0.55	0.30
Ca (%)	0.95	1.01	0.69
P (%)	0.92	0.78	0.58
Na (%)	0.19	0.17	0.15

^1^Data presented for starter diets were provided by the manufacturers without hay components analysis and may differ from actual composition. No own laboratory analyses were conducted for the starter feeds and alfalfa hay

^2^Metabolizable energy

The composition of alfalfa hay was not analysed in this study. The diets used in this study reflect common feeding practices, in which producers routinely supplement starter diets with chopped alfalfa or alfalfa hay.

The metabolizable energy content (ME) of the TS and CS were similar, whereas, the OS contained much less energy. Also, there were remarkable differences in the crude protein (CP), crude fiber (CF), and ash contents of the diets. The CP content of the TS was 8% higher than in the OS and CS. However, the OS had three times higher CF content than the TS and more than double that of the CS. Also, the ash content of the OS was 1.5 times higher than in the CS and higher than in the TS, and the same tendency could be presented for the lysine content of the TS.

### Description of age groups

We focused the experiment on the first 4 weeks of chick development, as farmers typically sell them at this age. Evaluating growth intensity and mortality during this period is crucial for assessing the economic impact of different feeding strategies. We identified each chick individually and weighed them at 5 days of age and then weekly for 4 consecutive weeks using an electronic scale with a precision of 2 decimals. We conducted the first weighing immediately after hatching and repeated the process every 7 days.

### Description of hatching groups

The incubation period for ostrich chicks ranges from 38 to 45 days (Hermes 1996), with the ideal hatching time occurring around day 42 (Minnaar 1998). In Hungary, healthy chicks typically begin breaking the eggshell on day 40. However, many are too weak to hatch on their own by this time and may not survive without assistance. In such cases, farmers use candling to locate the chick’s head inside the egg, then carefully create an air hole in the shell with a hammer. This allows the chick to breathe and hatch more easily. Assisted chicks usually hatch on day 41 or 42.

We classified the original groups of chicks (75 individuals altogether) into groups based on the hatching time (day 40, 41, or 42) and hatching condition (assisted or unassisted), as follows:

- hatched on day 40 without assistance (D40) (*n* = 19).
- hatched on day 41 with assistance (D41) (*n* = 34).
- hatched on day 42 with assistance (D42) (*n* = 22).

The groups will be referred to as “hatching groups” later in the text.

### Classification of mortality groups

We tracked mortality daily by collecting the dead individuals. We classified the birds according to their age in days. Chicks that died between 1 and 7 days of age were classified as week 1 mortality (W1M), those that died between days 8 and 14 were recorded as week 2 mortality (W2M), and deaths occurring after 14 days were classified as week 3 mortality (W3M). No deaths were observed on week 4.

### Evaluated parameters

- mean weekly weight (g) of chicks in their first 4 weeks of age,
- mean weekly weight (g) and weight gain (g/week) of chicks by feeding groups,
- mean weekly weight (g) and weight gain (g/week) of chicks by hatching groups,
- relative risk of chicks by feeding groups,
- relative risk of chicks by hatching groups.

### Statistical analysis

First, we analysed the normality of data distribution by applying the Kolmogorov-Smirnov and Shapiro-Wilk tests in Statistical Package for Social Sciences (SPSS 22.0). We used the inverse distribution function after performing fractional ranking of cases in SPSS for data normalization (standard transformation). We evaluated the effects of age, feeding, and hatching groups on chick weekly weights and weight gains using univariate analysis of variance under the framework of General Linear Model (GLM) in SPSS 22.0. Models were applied separately for weekly weight and weight gain analysis. In both models, age, feeding, and hatching groups served as fixed factors, while average weekly weights and weekly weight gains were dependent variables. We also investigated the correlations between hatching group and hatching weight, and hatching group and 4-week growth intensity with Pearson’s bivariate correlation in the SPSS 22.0 program. We used Post Hoc Test (Tukey’s HSD) to compare means, setting the significance level at *P* < 0.05. Figures present the mean and standard error of the mean (mean ± S.E.M.).

The effect of hatching and feeding groups on the relative culling risk of chicks was performed using the Kaplan–Meier procedure and Cox regression in SPSS 22.0 according to Baccouri and Posta (2025). The Kaplan–Meier method estimates time-to-event outcomes in the presence of censored observations by calculating the conditional probability of survival at each event time point and multiplying these probabilities to obtain the survival function. Cox regression was applied to assess the influence of predictor variables (covariates) on survival outcomes. This method provides estimated coefficients for each covariate, enabling the simultaneous evaluation of multiple factors and allowing the inclusion of continuous predictors. Risk ratios were reported for each factor relative to a reference category, which was defined as the group with the lowest estimated risk ratio.

## Results

### Mean weights and weight gains of chicks by age

**Fig. 1:**
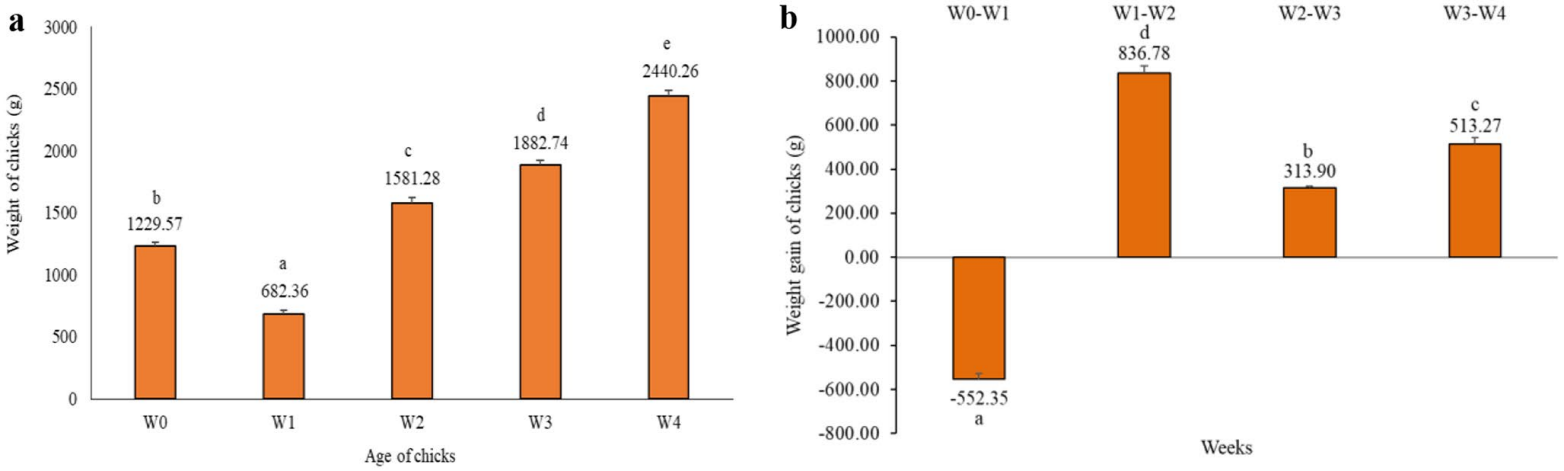
**a**,** b** Changes in the weekly mean weights and weight gains of ostrich chicks until 4 weeks of age. ^a, b^ Different letters indicate significant differences between age (*P* < 0.05). Error bars represent the S.E.M. (standard error of the mean)

The mean hatching weight of chicks (*n* = 75) was 1229 g, increasing to approximately 2500 g by 4 weeks of age. We found no differences in mean body weight among hatching groups at any age (Fig. 1a). Body weight decreased between W0 and W1 and increased from W2 onward. The highest weight gain occurred at 2 weeks of age (W1-W2), whereas the lowest gain was recorded on week 3 (W2-W3) (Fig. 1b).

### Weight and weight gain of chicks by feeding groups

**Fig. 2:**
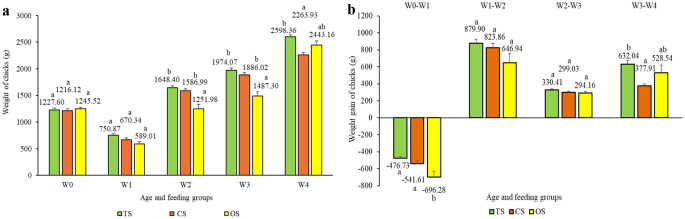
**a**,** b** Mean weights and weight gains of chicks by feeding groups during the first 4 weeks of age. ^a, b^ Different letters indicate significant differences within age (*P* < 0.05). Error bars represent the S.E.M. (standard error of the mean)

The effect of feeding group on the mean weight of chicks was significant between W2 and W4. The OS group showed lower values than TS and CS at W2 and W3, while at W4, the lowest mean weight occurred in CS and the highest in TS (Fig. 2a). At W1, we observed the greatest decrease in body weight gain in the OS group, whereas TS and CS did not differ from each other. At W4, we detected a significant difference in weight gain among groups, with CS showing the lowest and TS the highest mean weekly gain. At W2 and W3, TS and CS exhibited higher mean body weights than OS. We observed the greatest weekly decrease in body weight on the 1st week (W0-W1) in the OS group. The lowest average weekly gain (193.34 g) also occurred in OS, followed by CS (239.79 g), while TS showed the highest average weekly gain (341.41 g). We did not detect significant differences among groups in the subsequent weeks (Fig. 2b).

### Mean weight and weight gain of chicks by hatching groups

**Fig. 3:**
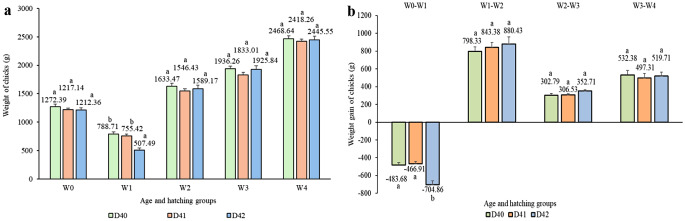
**a**,** b** Mean weights and weight gains of chicks by hatching groups during the first 4 weeks of age. ^a−e^ Different letters indicate significant differences within age (*P* < 0.05). Error bars represent the S.E.M. (standard error of the mean)

There was no difference in the mean weights of chicks between hatching groups during the 4-week period (Fig. 3a). Chicks in the D40 and D41 groups showed lower weight loss between W0 and W1 compared to those chicks in D42 group but groups did not differ in the subsequent weeks (Fig. 3b). We detected no correlation between hatching group and hatching weight, nor between hatching group and 4-week weight gain (*P* > 0.05).

### Relative risk of chicks by feeding and hatching groups

The effects of feeding and hatching groups on the relative risk of chicks in their first 4 weeks of age were both significant (Table 2).

**Table 2:** Significance values of the analysed factors for relative risk

	Feeding groups			Hatching groups		
	Chi-Square	df^1^	Sig.^2^	Chi-Square	df^1^	Sig.^2^
Log Rank (Mantel-Cox)	19.853	2	*P* < 0.01	26.643	2	*P* < 0.01
Breslow (Generalized Wilcoxon)	20.154	2	*P* < 0.01	25.327	2	*P* < 0.01
Tarone-Ware	20.026	2	*P* < 0.01	25.977	2	*P* < 0.01

^1^Degree of freedom

^2^Significance value

Beta (β) coefficients and Wald statistics for feeding and hatching groups are shown in Table 3.

**Table 3:** Risk statistics for feeding and hatching groups

Feeding and hatching groups	β	S.E.^1^ of β	Wald statistics	df^2^
TS			3.128	2
CS	0.700	0.568	1.521	1
OS	1.146	0.649	3.120	1
D40			9.397	2
D41	0.350	0.918	0.145	1
D42	1.798	0.755	5.676	1

^1^Standard error

^2^Degree of freedom

**Fig. 4:**
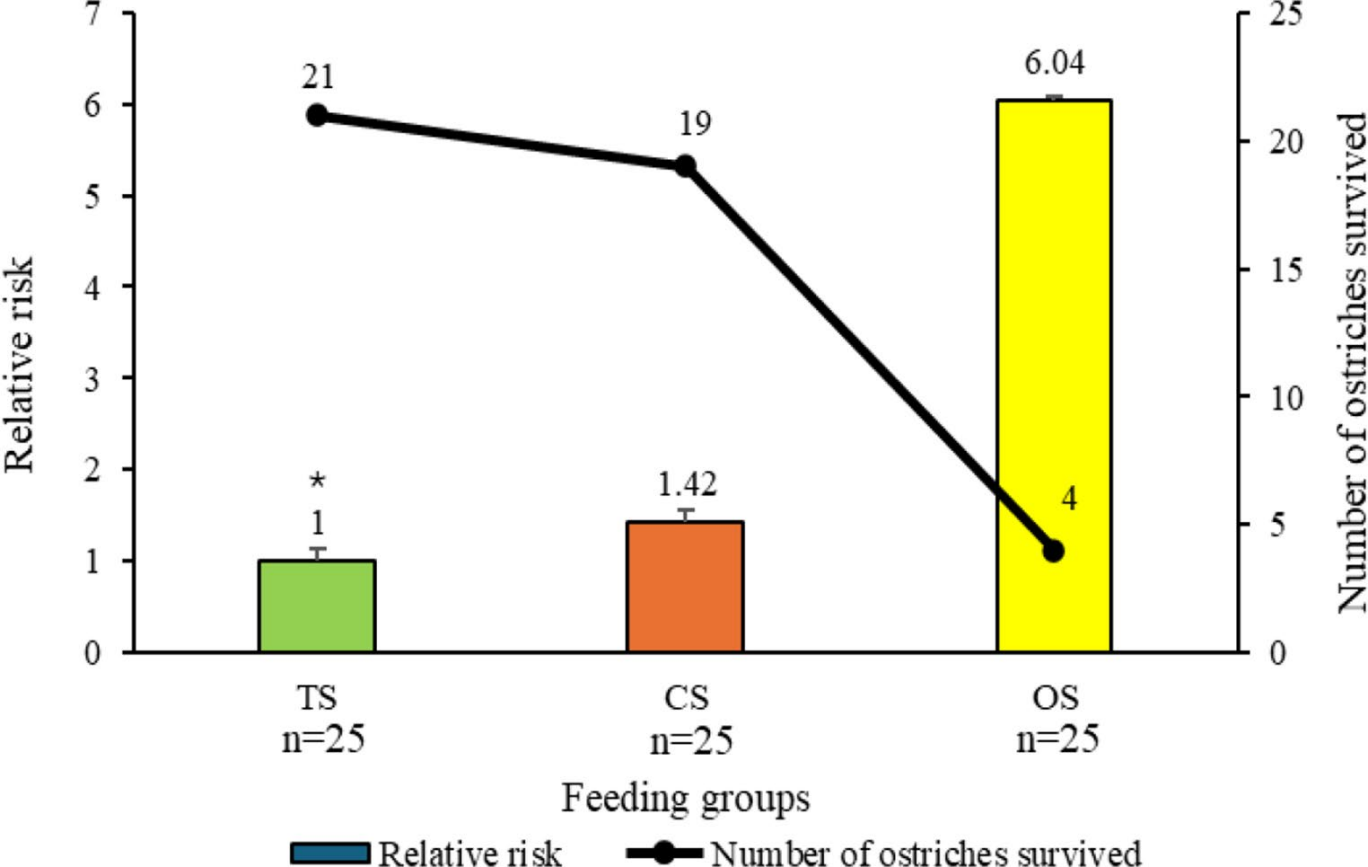
Relative risk of chicks affected by feeding groups during the first 4 weeks of age. The * indicates the reference group for Cox regression

**Fig. 5:**
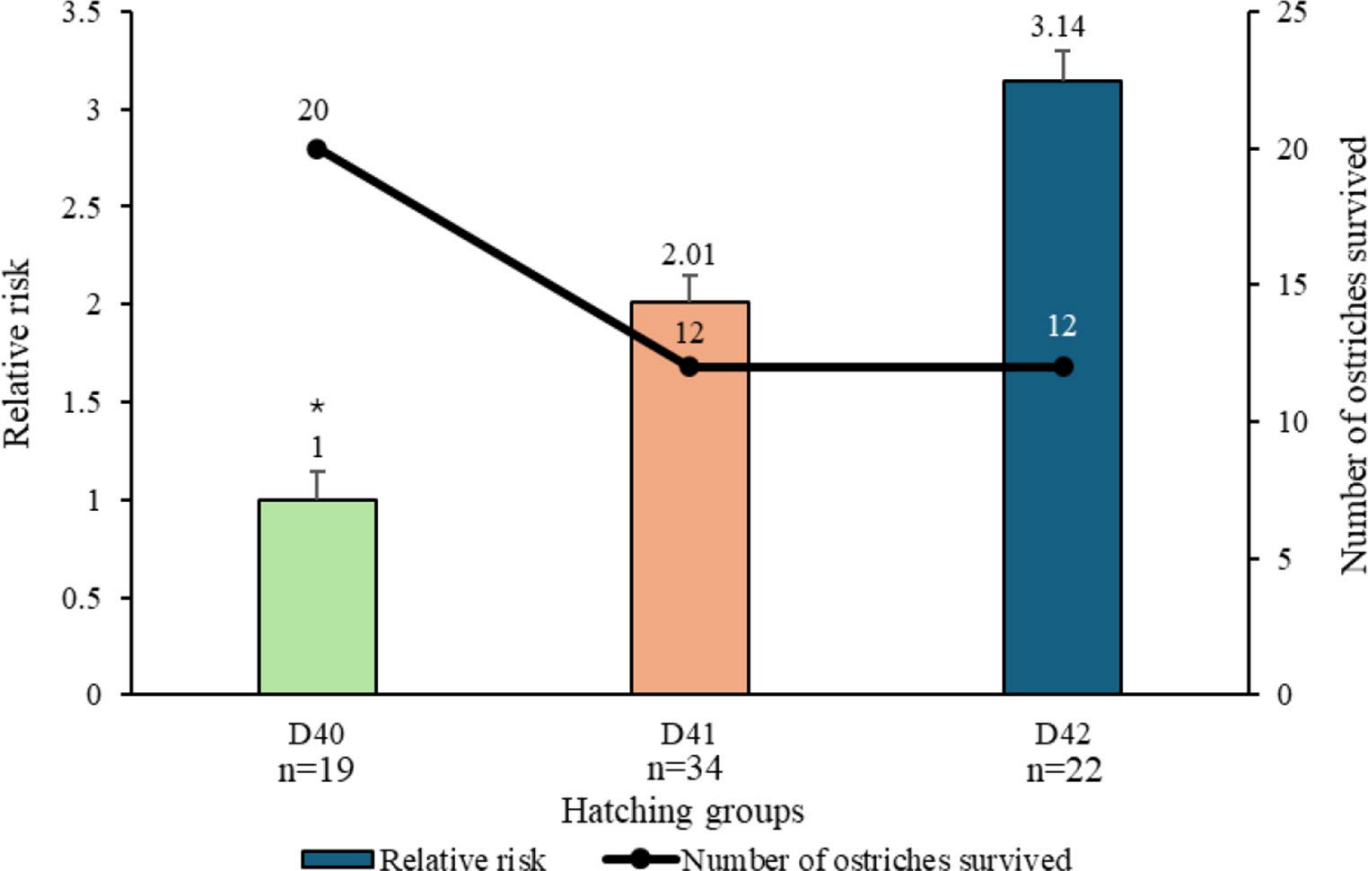
Relative risk of chicks affected by hatching groups during the first 4 weeks of age. The * indicates the reference group for Cox regression

The estimates of risk ratio and the number of animals survived are presented in Figs. 4 and 5 for feeding and hatching groups, respectively.

Regarding feeding groups, we observed the highest culling risk in the OS group, which was 6 times higher than in TS and 4.25 times higher than in CS, while CS did not differ from TS (Fig. 4). The OS group exhibited the lowest survival rate (16%, 4 chicks) during the 4-week experimental period. The CS group represented a 76% survival rate (19 chicks), while TS reached 85% (21 chicks). In total, 42 chicks survived until the age of 4 weeks, representing 58.67% of the initial population. Regarding age, we observed the highest survival rates at W1 and W4, with no mortality recorded. Survival was 95% at W3 and 64% at W2.

Among hatching groups, we found the highest culling risk in the D42 group, which was 3 times higher than in the D40, while D41 showed approximately twice the risk compared to D40 (Fig. 5). The D40 group exhibited the highest survival rate (80%, 20 chicks), whereas D41 and D42 showed similar survival rates, averaging 47% (12 chicks).

## Discussion

The present study aimed to evaluate how key nutritional characteristics of starter diets, rather than their commercial designation, influence early growth performance and survival of ostrich chicks during the first 4 weeks post-hatch. Although the diets applied in this experiment were commercially labelled for different poultry species, only one representative starter feed from each category was tested. Therefore, differences observed among feeding groups are interpreted throughout this discussion mainly on the basis of dietary composition, especially metabolizable energy (ME), crude protein (CP), and crude fibre (CF) content, and not as inherent effects of species-specific feed formulation. Early chick mortality and reduced growth are still major problems in ostrich production, and nutrition is generally considered one of the most important factors determining chick vitality in this sensitive period. By investigating starter diets commonly used in practice but differing markedly in energy density and nutrient composition, the present study provides information on which nutritional profiles can better adjust to the physiological requirements of young ostrich chicks.

### Weight changes and early nutrition

Brand (2014) stated that the optimal day-old weight for ostrich chicks would be between 800 and 850 g from the vitality point of view. Verwoerd et al. (1999) determined a higher interval in the optimal weight category who established that chicks weighing between 780 and 975 g at hatch had the best quality with low mortality. Also, Cloete et al. (2004) measured mean chick hatching weight 858 g. Compared to the international literature, we experienced much higher hatching (W0) weights indicating an appropriate breeding technology. Chick weight and therefore quality are influenced by many factors including genotype, parent age and nutrition, egg size, and hatching conditions (Brand 2014; Engelbrecht 2013). So, differences can be explained by any of the previously mentioned parameters which were not disclosed in the studies. Glatz and Miao (2008) experienced that generally, ostrich chicks lose 10% of their hatching weight until 5 to 7 days of age. However, in our case, it was more than 4 times higher than optimal. Engelbrecht (2013) also supported a decrease in chick ostrich weight in the first 4 days of life. This phenomenon could be explained by the absorption of yolk and subcutaneous oedema in the first few days, and the disability of eating right after hatching (Mushi et al. 1998). Cooper (2001) also declared that ostrich chicks not provided by feed immediately after hatching lose 1.35 g weight in every hour. In broilers, Chamblee et al. (1992) observed that the intensity of yolk absorption sharply increases within 48 h after hatching and the growth starts within the first 24 h. Besides the authors’ opinions, the weight loss between W0 and W1 could be resulted by the slower utilization of yolk due to the lack of feeding immediately after hatching. Information on the feeding of chicks regarding their earliest life period is controversial. According to Elobeid et al. (2014) and Kocan and Crawford (1994), ostrich chicks do not need feed and water on the 1st week. Also, Viljoen et al. (2012) observed that in ostrich, chick starvation accelerates yolk absorption. Conversely, several literature sources support the fact that newly hatched chicks need water and feed right after hatching both in the case of ostrich and chicken. For example, Verwoerd et al. (1999) and Deeming (1999) in ostrich and Noy et al. (1996) in chicken stated that chicks should be fed immediately after hatching to stimulate the development and operation of the digestive tract and yolk utilization. Otherwise, chicks will weaken and fade by the negative effects of fasting on their digestive tract and enzymes (Mushi et al. 2004). The international literature also suggests feeding green forages and feeds, such as finely chopped fresh alfalfa, vegetables, and green leaves. Besides the nutrient content, the green colour attracts chicks to consume the feed (Miao et al. 2003). It was also mentioned that pecking forage can reduce the presence of feather pecking by alleviating the boredom of birds (Christensen and Nielsen 2004).

### The role of specific nutrients (CP, ME, CF)

Based on the previously mentioned literature findings, it should be encouraged to feed chicks immediately after hatching to avoid significant weight loss and to support their health and subsequent development. In the present experiment, chicks showed a marked body weight decrease during the first week, followed by clearly diet-dependent growth patterns related to metabolizable energy, crude protein and crude fibre content.

Adewumi et al. (2017) evaluated the weight gain of 15 ostrich chicks between 1 and 6 weeks of age and reported continuous growth without weight decrease during the first week. In that study, chicks received a diet with relatively high metabolizable energy (13 MJ/kg) and moderately high crude protein content (24%). In comparison, in the present study, chicks fed a diet containing 11.4 MJ/kg metabolizable energy, 25.9% crude protein and 3.9% crude fibre achieved substantially higher growth rates, with an average weekly weight gain of 341.41 g, despite experiencing initial post-hatch weight loss. In Botswana, Mushi et al. (1998) investigated ostrich chicks and observed similar body weights at 4 weeks of age, although hatching weights were approximately 200 g lower. The diet used contained around 20% crude protein and was supplemented with *ad libitum* chopped alfalfa. In the present experiment, a diet with comparable crude protein level (17.9%), adequate metabolizable energy (11.1 MJ/kg) and moderate crude fibre content (5.08%) resulted in intermediate growth performance, with an average weekly weight gain of 239.79 g and a survival rate of approximately 76%, indicating that moderate protein intake can support acceptable growth when energy supply is sufficient and fibre levels are not excessive.

Compensatory growth is known in the literature in broiler chickens (Radulovic et al. 2021); however, it has not been documented in ostriches. This phenomenon is considered complex, involving nutritional, physiological, endocrine and metabolic factors. In the present study, no clear compensatory growth was observed during the first 4 weeks, as chicks exposed to nutritionally unfavourable conditions did not catch up later. Specifically, chicks fed a diet containing 8.0 MJ/kg metabolizable energy, 17.8% crude protein and 13.0% crude fibre showed the lowest average weekly weight gain (193.34 g) and extremely high mortality, with only 16% surviving to 4 weeks of age.

The most pronounced differences in growth performance and survival in the present experiment were therefore associated with the interaction of metabolizable energy, crude protein and crude fibre levels. Chicks receiving the diet with 25.9% crude protein, 11.4 MJ/kg metabolizable energy and 3.9% crude fibre showed the highest growth intensity (341.41 g/week) and the lowest cumulative mortality (< 10%). In contrast, the diet characterized by 17.8% crude protein, 8.0 MJ/kg metabolizable energy and 13.0% crude fibre resulted in severely impaired growth and cumulative mortality exceeding 80%. Carstens et al. (2014) provided pre-starter diets with different crude protein levels (16.80%, 20.28% and 23.48%) but uniformly high metabolizable energy content (14.5 MJ/kg) and low crude fibre levels (2.9–3.4%). Under these conditions, no significant differences in weight gain were observed during the first 6 weeks. Compared to those results, the present study indicates that when metabolizable energy supply is lower (8.0–11.4 MJ/kg) and crude fibre content is higher (up to 13.0%), protein utilization efficiency is markedly reduced, even at comparable crude protein levels. It has been reported that, before 3 months of age, ostrich chicks have a limited ability to digest fiber due to the underdevelopment of the large intestine and its microbiome (Angel 1993, 1996; Iji et al. 2003). In agreement with these findings, the high crude fibre content (13.0%) of one dietary treatment in the present study likely diluted metabolizable energy availability and contributed to poor growth performance and high mortality observed mainly between the second and third weeks of age. Genetic factors may also influence chick growth performance, although these were unknown in the present experiment. Nikravesh-Masouleh et al. (2021) reported that neither dietary protein nor energy level alone significantly affected weight gain, highlighting the importance of balanced dietary composition. Similarly, Azahan and Noraziah (2011) demonstrated higher body weights in ostriches receiving medium and high crude protein diets. In line with these studies, the present results demonstrate that crude protein can contribute to improved growth performance primarily when adequate metabolizable energy is available and crude fibre levels remain low, as reflected by the combination of 25.9% crude protein, 11.4 MJ/kg metabolizable energy and 3.9% crude fibre in the best-performing group.

### Hatching factors

Even though chicks in group D41 were the closest to the ideal maximal weight loss which is generally 10% according to Glatz and Miao (2008), we experienced 4 to 6 times higher losses (38% to 58%). The authors did not mention but 10% loss was probably the case in post-hatch fed chicks. Also, Brand et al. (2024) stated a 3% to 4% weight loss from hatching to 2 days of age due to an excessive moisture loss by lung ventilation, yolk absorption and faecal discharge. It is considered a satisfying extent of weight loss since chicks were *ad libitum* fed immediately after hatch. Based on hatching conditions in ostrich, Brand et al. (2024) established 12.4% higher weight gains in unassisted chicks than in chicks with cracked eggshell and 24.6% higher weight gains than in chicks with eggshell removed after external pipping until 147 days of age. The study highlighted that natural hatch was more favourable than human intervention during hatching. A similar trend was observed in our study; however, the smaller sample size in our evaluation may have limited the statistical significance of the results. A larger population, such as the 254 individuals analysed by Brand et al. (2024), would likely enhance the robustness and generalizability of our findings. International literature dealing with this topic in ostrich is limited, however there is abundant information on broiler chickens. In Bovans hybrid, hatch window (24 to 48 h) is reported to affect post-hatch growth intensity. The authors found negative correlations between hatching time and weight gain from day 4 after hatching during the 56-day experiment in females and until 10 days post-hatch in males. Similar to our findings, hatching time did not influence the hatching weight of broiler chicks (Løtvedt and Jensen 2014). In agreement with our findings, neither Careghi et al. (2005) established a correlation between the hatch window and hatching weight of Cobb broiler chicks. Conversely, in Ross-308, the hatching weight and weight at 3 days of age were higher in early hatched birds compared to the mid-term and late-hatched ones, however, there was no difference between the body weights from day 10 (Boyner et al. 2021). In contrast to our findings, Van de Ven et al. (2011) in broiler chicks found that late hatchers (incubation time (IT): 493 h) had lower weights on day 7 and 21 compared to mid-term (IT: 480 h) and early (IT: 465 h) hatchers. Tona et al. (2003) and Careghi et al. (2005) in broiler chicks also stated that egg weight at set, chick weight at hatch, and hatch window are strongly correlated parameters. Higher egg weights result in heavier chicks that hatch later than lighter chicks with smaller initial egg weights. However, it was not supported by our results in ostriches since hatching condition was not correlated with hatching weight, neither we had data on initial egg weights.

### Mortality

Reported mortality rates for farmed ostrich chicks vary widely, ranging from 10% to 90%, depending on the country, farm conditions, and age of birds (Ashash et al. 1996; Deeming et al. 1993; Deeming and Ayres 1994). Cloete et al. (2002) reported that mortality within the first 4 weeks accounted for approximately 60% of total losses observed by 90 days of age. Their findings indicated a sharp increase in mortality during the first 6 days, followed by a rapid decline between days 11 and 13. Cumulative mortality by 28 days of age was 46.7%, which aligns closely with the present study. The authors identified hatching month, incubator type, and day-old chick weight as influential factors in early chick survival; however, these parameters were uniform across all chicks in the current study. In their study, they identified the critical hatching weights between 687 and ≥ 762.5 g within which 40% to 70% did not survive. Also, they (Cloete et al. 2002) found remarkable mortalities for chicks with average weight of 1050 g at 28 days of age. Both established hatching weights and 28-day-old weights were extremely low, approximately half the corresponding values recorded in our study. Also, they established that the mortality decreased to 20 to 30% for chicks with weights > 1950 g which is still lower than the one we observed at the same age. The relationship between hatching weight and mortality was not evident in our evaluation, as chicks with a wide range of initial weights (956 g to 1384 g) succumbed at both 2 and 3 weeks of age. Deeming et al. (1993) documented a 19% mortality rate in chicks up to 5 weeks of age in Great Britain, a figure comparable to that observed in the TS group of this study. The literature does not provide a definitive cause for early chick mortality. In the present study, the elevated mortality observed from the second week onward coincided with the application of starter diets differing markedly in metabolizable energy, crude protein and crude fibre content. These findings suggest that differences in dietary composition may have contributed to survival outcomes during this critical period, in agreement with previous observations emphasizing the importance of early nutritional conditions (Mushi et al. 2004; Noy et al. 1996). According to Cooper (2001), a common misconception among ostrich breeders is that, after day 42 of incubation, manual shell breaking is necessary to prevent chick hypoxia. However, Cooper argues that such intervention may be detrimental, potentially harming the chicks. In our study, while we do not believe that chicks were physically harmed by shell assistance, those that required help were likely to have had reduced vitality and insufficient strength to hatch independently. Without assistance, such weaker chicks would likely not have survived. Given their high market value, intervention may be economically justified, but biologically, it suggests inferior viability. Our findings support the conclusions of Cloete et al. (2002, 2012), who reported that assisted chicks exhibited higher early mortality compared to those that hatched unassisted. This trend may be explained by the superior inherent vitality of independently hatched chicks. This hypothesis is further supported by Deeming and Ayres (1994) and Cooper (2001), who noted that hatch assistance often results in weak, lower-quality chicks, contributing to a higher mortality rate among late-hatched and assisted individuals. Cloete et al. (2002) categorized chicks based on the day of external pipping (< 41 days, 41–41.9 days, and > 42 days). Although they did not find a statistically significant correlation between pipping day and mortality, a trend toward higher mortality in both early and late pipping groups was observed. More recently, Brand et al. (2024) classified chicks according to hatching day (41, 42, or 43) and level of assistance. Their study also concluded that mortality was independent of hatching conditions. However, earlier research by Kingston (1979) on broiler chicks reported higher mortality in late-pipping chicks (those hatching at 21 days and 12 h) compared to those pipping earlier (19 or 20 days). Additionally, early-hatched chicks were more prone to dehydration, while late-hatched chicks more frequently exhibited leg deformities, highlighting the complex relationship between hatching time, developmental issues, and post-hatch survival.

### Limitations and practical implications

While this study provides meaningful insights into the effects of age, feed nutritional composition and hatching condition on the weight gain of young ostriches, some limitations should be acknowledged.

First, own laboratory analyses were not conducted on the analytical nutritional composition of starter diets therefore conclusions could be made only based on the investigations of the manufacturer. Furthermore, the analytical composition of the alfalfa hay was not analysed even though it made up almost half of the daily feed amount. These limitations restrict the accurate estimation of total nutrient intake and the potential variation in quality may have contributed to the differences among groups.

The second limiting factor was the lack of repetition in the case of batches for each diet type which limits the ability to account for potential batch-to-batch variability in nutrient composition and to draw long-term conclusions on the dietary effects observed. The absence of batch replication further limits the generalizability of the findings to other feed batches or formulations of similar types.

Third, dietary treatments may have been confounded with unmeasured factors such feed palatability, physical form, or feed presentation. These factors influence voluntary feed intake, however, were not quantitatively assessed in this study. Additionally, feed intake was not measured, and therefore differences in consumption, rather than feed composition alone, cannot be excluded as contributing factors.

Fourth, the small sample size on the last week of the experiment (*n* = 4 at W4) in the OS group may distort and reduce the statistical power and limits the robustness of conclusions on the late-stage growth performance of this group.

Finally, genetic factors may also influence chick growth performance; however, these factors were not characterized in the present study, representing a potential limitation of the findings.

## Conclusions

This study demonstrates that the nutritional composition of starter diets, particularly metabolizable energy, crude protein and crude fibre content, plays a critical role in the early survival and growth of ostrich chicks. The results indicate that differences observed between feeding groups were related to dietary characteristics rather than to the commercial designation of the feeds, as only one representative starter diet from each category was evaluated. Under the conditions of the present experiment, a starter diet with higher metabolizable energy (11.4 MJ/kg), high crude protein content (25.9%) and low crude fibre level (3.9%) yielded the best survival (85%) and growth. In contrast, diet with low metabolizable energy (8.0 MJ/kg) and protein content (17.7%), whereas high crude fibre content (13.0%) resulted in weak performance and only 16% survival. A diet with intermediate nutritional values (11.1 MJ/kg metabolizable energy, 17.9% crude protein and 5.08% crude fibre) led to moderate performance and culling risk. Hatching assistance did not show a statistically significant effect on 4-week chick weight gain. However, we established the lowest culling risk in the unassisted group.

The findings suggest that insufficient energy supply and excessive crude fibre during the starter phase may substantially impair early chick viability, while higher crude protein levels appear beneficial only when adequate energy density and low fibre content are ensured. From a practical perspective, the results highlight the importance of prioritizing metabolizable energy density, appropriate crude protein level and low crude fibre content when selecting starter diets for ostrich chicks, irrespective of feed labelling.

## Data Availability

The data presented in this study are available on request from the corresponding author. The data are not publicly available due to privacy concerns.
